# The Ashtamudi Lake short-neck clam: re-assigned to the genus *Marcia* H. Adams & A. Adams, 1857 (Bivalvia, Veneridae)

**DOI:** 10.3897/zookeys.799.25829

**Published:** 2018-11-28

**Authors:** Anitha R. Arathi, P. Graham Oliver, Raveendhiran Ravinesh, Appukuttannair Biju Kumar

**Affiliations:** 1 Department of Aquatic Biology and Fisheries, University of Kerala, Thiruvananthapuram- 695581, Kerala, India University of Kerala Kerala India; 2 National Museum of Wales, Cathays Park, Cardiff, CF10 3NP, UK National Museum of Wales Cardiff United Kingdom

**Keywords:** Fisheries management, India, *
Marcia
*, MSC certification, *
Paphia
*, *
Protapes
*, Venerid clam, Veneridae

## Abstract

The economically valuable bivalve mollusc, known as the short-neck clam, is the major fishery resource of the brackishwater Ashtamudi Lake in Kerala, India. This fishery carries a Marine Stewardship Council certification for sustainability wherein it and all hitherto published reports identify the short-neck clam or yellow-foot clam as *Paphiamalabarica* (Dillwyn, 1817). It is noted that this name does not conform with current nomenclature and is now correctly referred to *Protapesgallus* (Gmelin, 1791). Furthermore, it is shown that the identification is also incorrect. Comparative shell morphology of venerid clams of the subfamily Tapetinae from the south Indian coast demonstrates that the short-neck clam in Ashtamudi Lake is *Marciarecens* (Holten, 1802). Small numbers of *M.opima* (Gmelin, 1791) were found in Ashtamudi Lake but appear not to be part of, or contribute significantly to, the fishery. The venerid clams *Protapesgallus* and *P.ziczac* (Linnaeus, 1758) are not found in Ashtamudi Lake but are inhabitants of the shallow coastal waters of south India. Descriptions of the four confused species *M.recens*, *M.opima*, *P.gallus*, and *P.ziczac* are given. On the basis of this study, the species involved in Marine Stewardship Council (MSC) certification may be better considered at the generic level of *Marcia* or at the species level as *Marciarecens*, the most dominant species in the Ashtamudi Lake clam fishery zone.

## Introduction

India supports extensive bivalve fisheries, notably for mussels, oysters, and clams, with an estimated annual production of 84,483 tonnes (CMFRI 2017). Clams form a subsistence fishery in Indian coastal waters, lakes, and estuaries, with a potential yield of 113,189 tonnes and the export from India is dominated by the short-neck or yellow-foot clam. A major part of this export is sourced from the Ashtamudi Lake in Kerala state, a designated Ramsar wetland on the southwestern coast of India. The Ashtamudi Lake is a large, basin-shaped estuary, some 62 km^2^ in area and discharging into the Laccadive Sea through a narrow channel less than 300 m wide (Mohamed et al., 2013). This estuary provides livelihoods for hundreds of people involved in clam fishing, preparation and packing (CMFRI 1988, 2015, 2017, Appukuttan 1993, 2016, Appukuttan et al. 1999, Mohamed et al. 2013). With proper management interventions the sustainability of the Ashtamudi clam fishery has been ensured (Mohamed et al. 2013, Appukuttan 2016) and has, since 2014, been certified under the eco-labelling scheme of the Marine Stewardship Council (Wakamatsu and Wakamatsu 2017). All published reports of this clam, from the Ashtamudi Lake, refer to it as *Paphiamalabarica* (Dillwyn, 1817), (Achari 1986, Kripa et al. 2006, CMFRI 2011, 2015, 2017, Appukuttan 2016). This name continues to be used despite the fact that it is a junior synonym of *Protapesgallus* (Gmelin 1791) (MolluscaBase 2018a).

During a bivalve training workshop in Kochi in 2016 (Nandan et al. 2016), further suspicions were raised about the taxonomy of clams brought from Ashtamudi Lake. The specimens at hand were supplied as short-neck clams but the shape and pallial sinus suggested that they did not belong to the genera *Paphia* or *Protapes* but to a different genus of the Tapetinae. This paper reports on a morphological analysis of the clams fished from the Ashtamudi Lake, the subsequent comparison of the shell morphologies of southern Indian Tapetinae and the correct identification of the Ashtamudi Lake short-neck clam.

## Materials and methods

**Figure 1.**
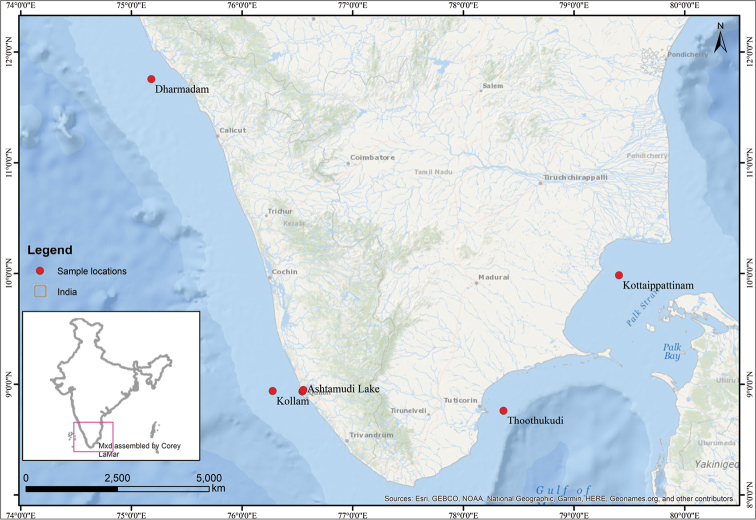
Sampling locations of venerid clams from the coast of southern India.

**Figure 2.**
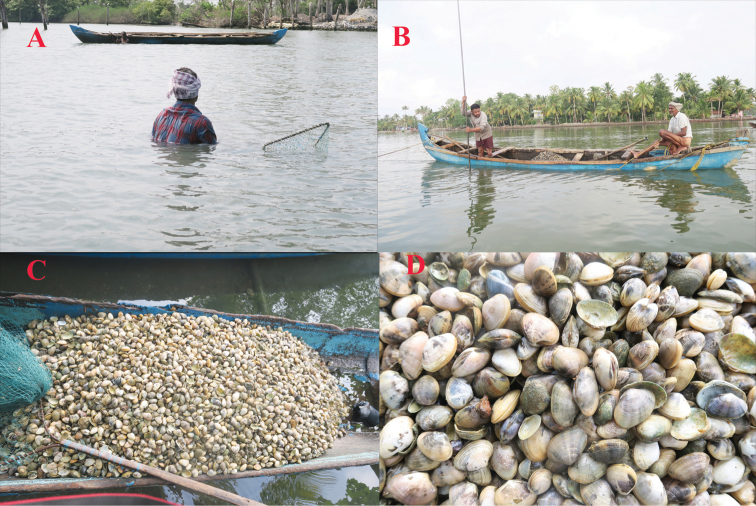
Small scale clam fishery in Ashtamudi Lake, Kerala, India **A** collecting by hand picking **B** collecting using a dredge net **C, D** a typical catch of clams.

In order to record the species diversity represented in clam fisheries, surveys were conducted in the clam fishing zones of Ashtamudi Lake (8°56'N, 76°30'E), during 2015–2017 (Figure 1). Specimens were collected by fishermen using hand dredge nets and hand picking (Figure 2). Over 200 specimens were procured in order to assess the variation and species diversity. Specimens were also collected from the shallow waters of the Tuticorin (Thoothukudi) coast, Tamil Nadu (Figure 1), which is the type locality for *Marciarecens* (Holten, 1802). Shallow offshore sampling on both southwestern and southern-eastern coasts of India was undertaken to collect clams of the genus *Protapes*. The specimens were collected by bottom trawlers at an average depth off 15–30 m off the Kollam and Kannur coastal regions of Kerala, India (Figure 1). The voucher specimens are deposited in the museum of Department of Aquatic Biology and Fisheries, University of Kerala, Trivandrum, Kerala, India (DABFUK).

An initial review was undertaken by examining literature, primarily that of Subba Rao (2017), but also of Huber (2010), for Tapetinae known from southern India.

The nomenclature was reviewed from all original sources including Gmelin (1791), Chemnitz (1795), Holten (1802), Dillwyn (1817), and Lamarck (1818). The taxonomic identification of the specimens was carried out using Fischer-Piette and Metivier (1971), Oliver (1992), Oliver and Glover (1996), Huber (2010), Ramakrishna and Dey (2010), and Subba Rao (2017). Synonyms were accepted from MolluscaBase (2018) (http://www.molluscabase.org).

Shell measurements such as shell length (maximum distance from anterior to posterior margin), shell height (maximum distance from dorsal to ventral margin), and shell breadth (maximum inflation of the valves when joined) were recorded based on Oliver and Glover (1996), to the nearest 0.1 mm using a digital Vernier calliper.

## Institutional abbreviations

**CMFRI** Central Marine Fisheries Research Institute, India

**DABFUK** Department of Aquatic Biology and Fisheries, University of Kerala, Trivandrum, Kerala, India

**MHNG** Geneva Museum of Natural History

**NMW** National Museum of Wales, Cardiff

## Results

### Identity of the Ashtamudi clam

Subba Rao (2017) records 17 species of Tapetinae from Indian waters but only two species were collected from the Ashtamudi Lake. Both species have a shell with no radial sculpture and this excludes the genera *Ruditapes*, *Venerupis*, and *Irus*. The outline is subovate with rounded lateral margins and this excludes the rhomboidal form of *Tapes* species that occur in India, as well as the trigonal *Macridiscus*. The pallial sinus in both Ashtamudi species is horizontally aligned and this excludes the genera *Paphia* and *Protapes*. The Ashtamudi clams therefore fall into the genus *Marcia*. Subba Rao (2017) records four species of *Marcia* from Indian waters, two with weak commarginal sculpture (*M.recens* Holten, 1802 and *M.opima* Gmelin, 1791) and two with prominent commarginal riblets (*M.hiantina* Lamarck, 1818 and *M.japonica* Gmelin, 1791) Both Ashtamudi clams have a weak commarginal sculpture and can be identified as *M.recens* and *M.opima*. Specimens collected from the coast at Tuticorin can also be identified as *M.recens*.

No specimens referable to the genera *Paphia* or *Protapes* were found among the Ashtamudi Lake samples, but were collected from the offshore sampling. Subba Rao (2017) recorded two species of *Protapes* but Huber (2010) noted a third under the name of *P.ziczac* (Linnaeus, 1758). Both *P.ziczac* and *P.gallus* (Gmelin, 1791) were collected from offshore sampling. As detailed below, the comparative shell morphology demonstrates that the Ashtamudi clam fishery is not based on *Paphiamalabarica* (= *Protapesgallus*), but primarily on *Marciarecens*. *Protapes* species are present around southern India but are absent from the Ashtamudi Lake, preferring open coastal waters. *Marciarecens*, by contrast, is widely distributed in estuarine and backwater habitats on both east and west coasts of India along with *M.opima*. Given the historical confusion we describe the species of *Marcia* in detail and give comparative descriptions of *Protapesgallus* and *P.ziczac*.

### Descriptions

#### Family Veneridae Rafinesque, 1815

##### Subfamily Tapetinae Gray, 1851

###### Genus Marcia

Taxon classificationAnimaliaVeneridaVeneridae

H. Adams & A. Adams, 1857

####### Type species.

*Venusopima* Gmelin, 1791

####### Description.

Moderately sized, outline triangular-ovate to elongate-ovate. Hinge with three cardinal teeth in each valve; posterior and middle cardinal bifid in left valve, middle cardinal bifid in right valve; laterals absent. Ligament external, elongate. Pallial sinus moderately deep, horizontally aligned. Sculpture variable from smooth to commarginal lines to weak commarginal ridges. Often highly and variably patterned externally with bold geometric blotches and radial rays. Inner shell margins smooth.

####### Remarks.

The species within the genus *Marcia* are rather variable in form with the sculpture varying from almost smooth (*M.opima*) to finely ridged (*M.japonica* and *M.hiantina*). Huber (2010) notes this variability and discusses, but rejects, the use of *Hemitapes*, Römer, 1864 for these more coarsely sculptured forms. All have a horizontally aligned pallial sinus and this contrasts with the steeply ascending orientation seen in *Protapes*. Furthermore in *Protapes* the sculpture is stronger with commarginal raised ridges; the posterior margin is obliquely truncated and the anterior pronounced with a depressed lunule.

###### Marcia opima

Taxon classificationAnimaliaVeneridaVeneridae

(Gmelin, 1791)

Figure 3

####### Original combination.

*Venusopima* Gmelin, 1791

####### Synonyms.

(from MolluscaBase 2018b) *Venuspinguis* Chemnitz, 1782 (unavailable), *Venusnebulosa* Gmelin, 1791; *Venustriradiata* Gmelin, 1791; *Venusgravida* Röding, 1798; *Tapesceylonensis* G. B. Sowerby II, 1852.

####### Type locality.

As the name *Venuspinguis* Chemnitz, 1782 is unavailable, this species takes the name of *Venusopima* Gmelin, 1791, both names referring to Chemnitz, 1782 tab. 34, figs 355–357. The type locality is given as East Indian Seas by Chemnitz (1782) but as India by Gmelin (1791).

####### Material examined.

Ashtamudi Lake, Kerala, 21 live collected specimens + 26 articulated conjoined valves.

####### Measurements.

Length 30.4–48.6 mm, mean L/H = 1.3, mean L/B = 1.6.

####### Description.

Shell equivalve, relatively thin; inflated, umbos prominent. Outline triangularly subovate, inequilateral, beaks slightly in front of midline. Lunule well defined, prominent, flattened, and broad. Escutcheon weakly defined. Shell surface smooth, glossy with faint growth lines. Adductor muscles of equal size. Pallial sinus horizontally aligned, broadly rounded, extending to midline of shell. External colouration variable and variously patterned, shades of brown, cream and dark grey with 3–4 radial darker bands. Internal colouration white.

####### Distribution.

*Marciaopima* is distributed throughout the Indian Ocean from the Red Sea to Indonesia. Authentic records are from East Africa, Kenya, Djibouti, Yemen, Arabian Gulf, Oman, Pakistan, India, Sri Lanka, Myanmar, Andaman Sea, West Malaysia, Penang, and Sulawesi (Huber 2010).

####### Remarks.

*Marciaopima* was originally described from India and it has a wide distribution on the east and west coasts of India including the Andaman-Nicobar and Lakshadweep islands (Ramakrishna and Dey 2010, Subba Rao 2017). A fishery for this species in the Ashtamudi Lake was reported by Appukuttan et al. (1985) but we cannot confirm the actual identity of the species involved. Other fisheries of this species are recorded by Subba Rao (2017), in particular at Ratnagiri (west coast) and Adyar River (east coast).

**Figure 3.**
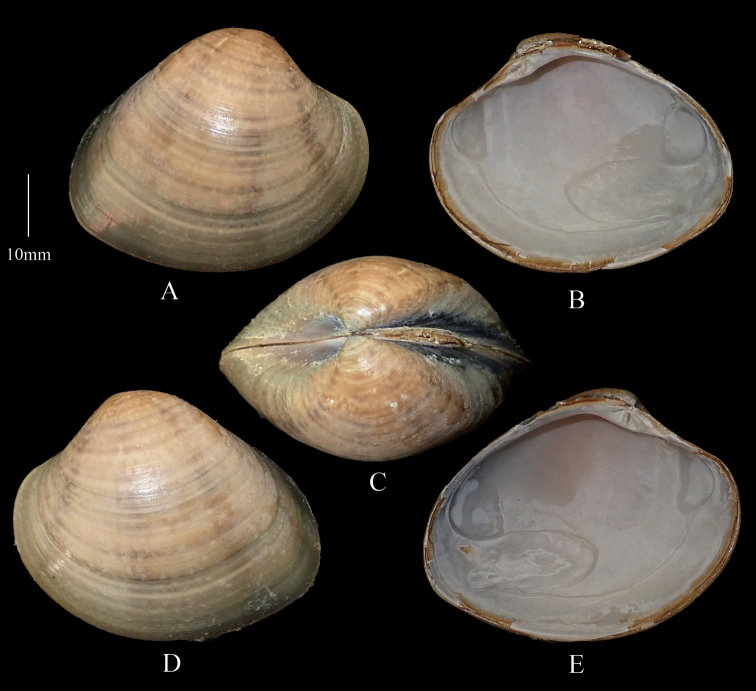
*Marciaopima***A** right valve dorsal **B** right valve ventral **C** dorsal margin **D** left valve dorsal **E** left valve ventral (DABFUK), Ashtamudi, Kerala.

###### Marcia recens

Taxon classificationAnimaliaVeneridaVeneridae

(Holten, 1802)

**Figure 4.**
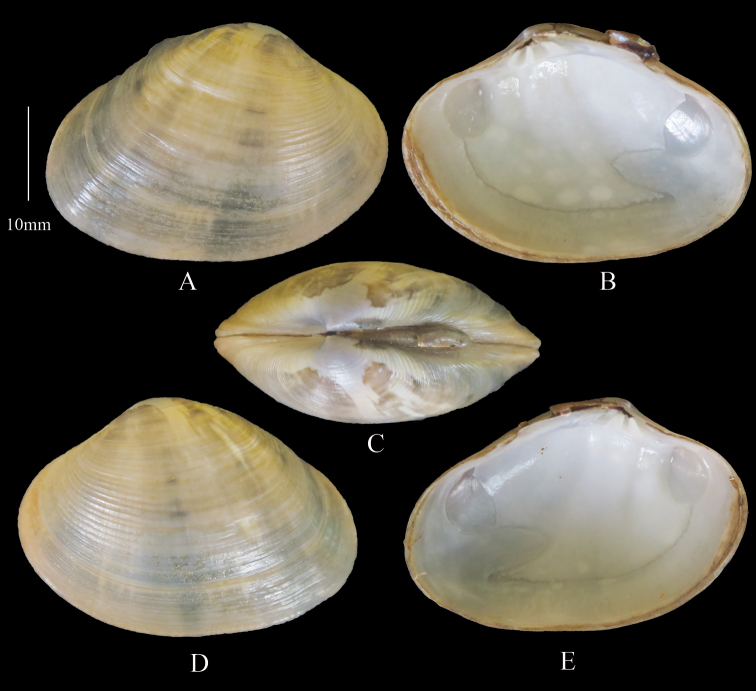
*Marciarecens* Topotype **A** right valve dorsal **B** right valve ventral **C** dorsal margin **D** left valve dorsal **E** left valve ventral (DABFUK), Tuticorin, Tamil Nadu.

**Figure 5.**
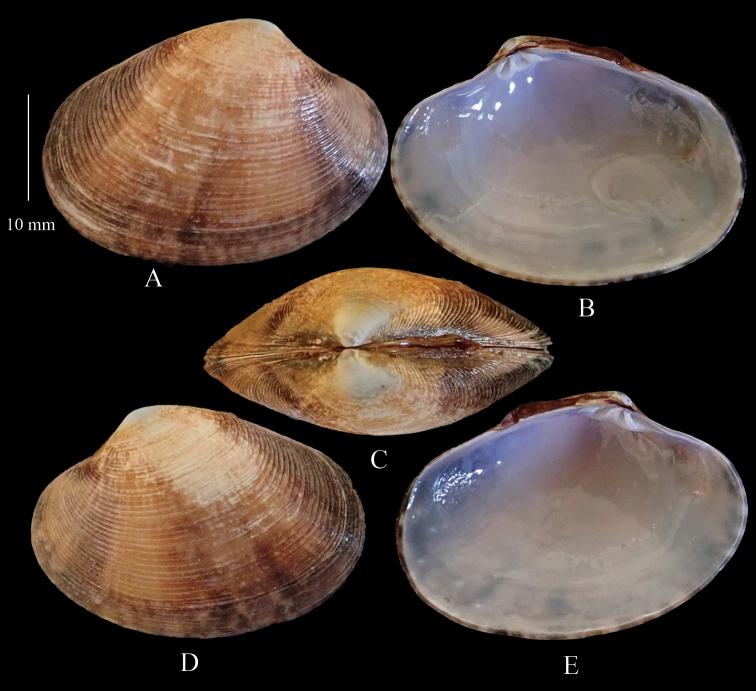
*Marciarecens* Morphotype 1 **A** right valve dorsal **B** right valve ventral **C** dorsal margin **D** left valve dorsal **E** left valve ventral (DABFUK), Ashtamudi Lake, Kerala.

**Figure 6.**
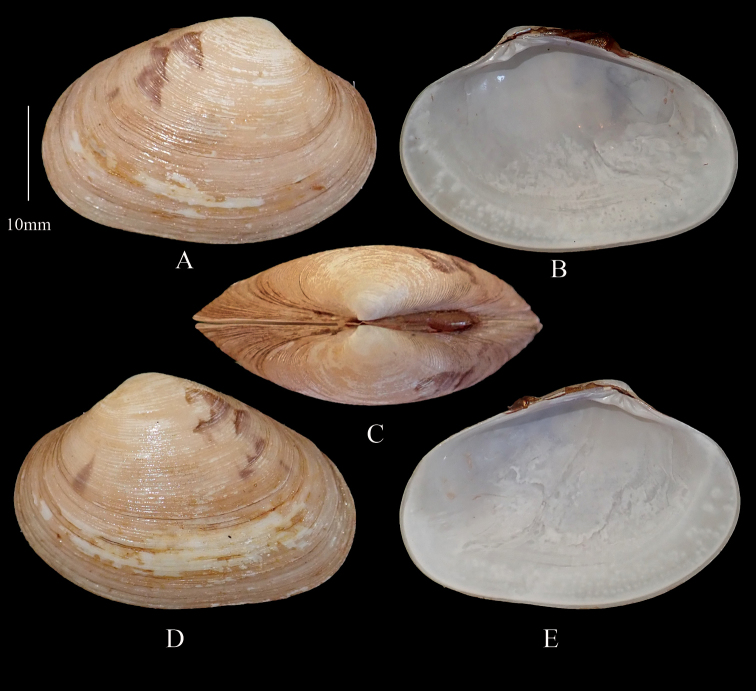
*Marciarecens* Morphotype 2 **A** right valve dorsal **B** right valve ventral **C** dorsal margin **D** left valve dorsal **E** left valve ventral (DABFUK), Ashtamudi Lake, Kerala.

**Figure 7.**
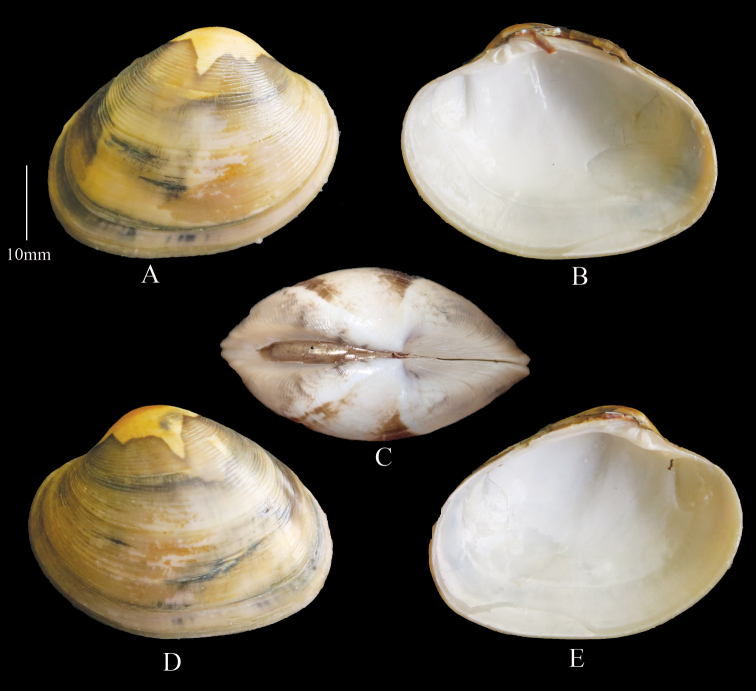
*Marciarecens* Morphotype 3 **A** right valve dorsal **B** right valve ventral **C** dorsal margin **D** left valve dorsal **E** left valve ventral (DABFUK), Ashtamudi Lake, Kerala.

**Figure 8.**
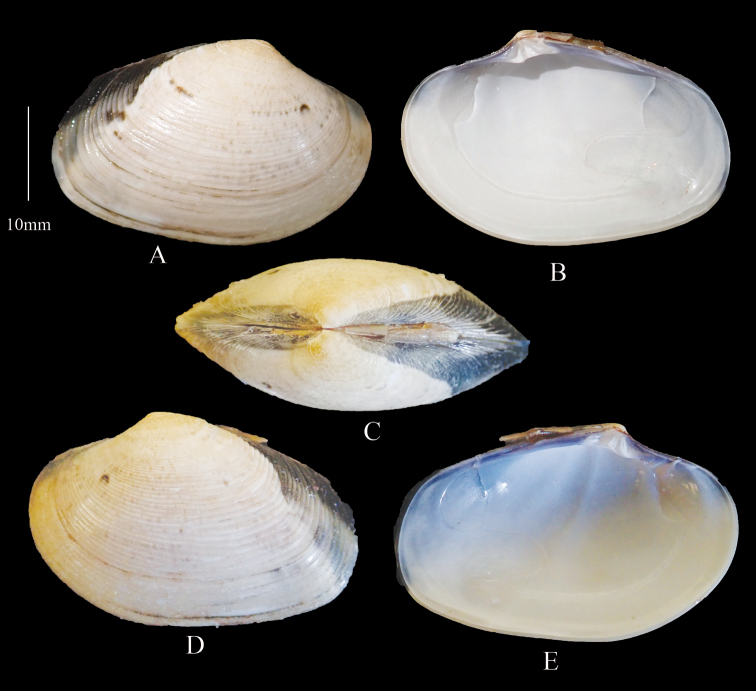
*Marciarecens* Morphotype 4 **A** right valve dorsal **B** right valve ventral **C** dorsal margin **D** left valve dorsal **E** left valve ventral (DABFUK), Ashtamudi Lake, Kerala.

**Figure 9.**
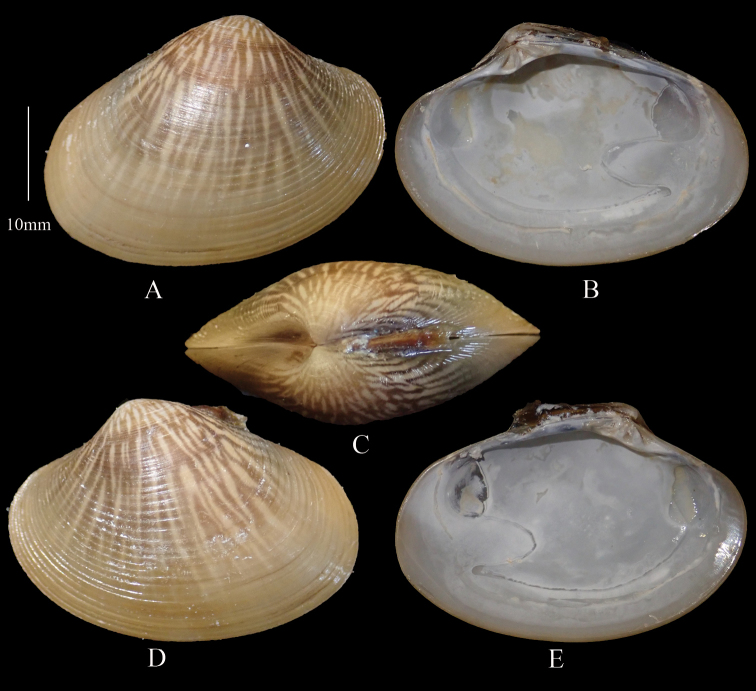
*Marciarecens* Morphotype 5 **A** right valve dorsal **B** right valve ventral **C** dorsal margin **D** left valve dorsal **E** left valve ventral (DABFUK), Ashtamudi Lake, Kerala.

Figures 4 , 5 , 6 , 7 , 8 , 9

####### Original combination.

*Venusrecens* Holten, 1802

####### Synonyms.

(from MolluscaBase 2018c) *Venusmarmorata* Lamarck, 1818; *Venusinterrupta* Koch in Philippi, 1849; *Tapeslaterisulca* G. B. Sowerby II, 1852; *Tapesbicolorata* Reeve, 1864; *Tapesferruginea* Reeve, 1864; *Tapesoccidentalis* Reeve, 1864; *Tapesorientalis* Reeve, 1864; *Tapessinensis* Reeve, 1864; *Hemitapesdohrni* Römer, 1870; *Tapesexserta* Römer, 1872.

####### Type locality.

Chemnitz (1795: 229) gives the type locality as Tuticorin on the Coromandel coast.

####### Material examined.

Tuticorin, 42 live collected specimens: Ashtamudi Lake, 217 live collected specimens: Mumbai (Bombay), Maharashtra, 6 empty articulated shells, as *Tapesmarmorata* Lamarck, leg. J. C. Melvill, NMW 1955.158: Thalassery (Tellicherry), Northern Kerala, 4 empty articulated shells, as *Hemitapesmarmorata* Lamarck, coll. H. C. Winckworth, 1931, NMW. 1955.158

####### Measurements.

Shells from Ashtamudi Lake and Tuticorin ranged in length from 12 to 51 mm. More detailed measurements are given for the morphotypes described below.

####### Description.

Shell robust, moderately thick, moderately inflated. Outline elongate subovate, inequilateral, beaks in front of midline. Lunule flattened, not well defined. Escutcheon weakly defined. Shell surface slightly glossy; sculpture commarginal, of weak lines and growth stops, some with more defined ridges especially over anterior area. Muscle scars weakly heteromyarian, posterior larger. Pallial sinus horizontally aligned, broadly rounded extending to one third of shell length. External colouration highly variable and variously patterned, cream, red, white or brown and patterned with 3–4 black radiating rays, or darker trigonal blotches over a light ground or with anastomosing narrow radial rays. Internal colouration white, some with pinkish umbonal cavity.

***Variability*** The type locality of *M.recens* is given as Tuticorin but without any further precision. Shells collected for this study from Tuticorin can be considered to come from the type locality and are given topotype status.

***Topotypes*** (Figure 4) Outline ovate-elongate. Yellowish brown with darker radial bands and umbonal blotching. Sample size 30 shells. Shell length range 31.2–54.4 mm, mean L/H = 1.3, mean L/B = 2.1.

Shells from Ashtamudi Lake show considerable variation in shell colour and pattern and these are defined as follows.

***Morphotype 1*** (Figure 5). Outline ovate–elongate. Reddish brown to light brown shells with dark brown to black radial rays. Sample size 82 shells. Shell length 15.4–45.8 mm, mean L/H = 1.4, mean L/B = 2.3.

***Morphotype 2*** (Figure 6). Outline ovate–elongate. White to cream shells with sparse blotching, lacking dark radial rays Sample size 75 shells. Shell Length 16.0–51.0 mm mean L/H = 1.4, mean L/B = 2.4.

***Morphotype 3*** (Figure 7). Outline trigonal–ovate, inflated. Yellowish brown shells with irregular sparse blotching. Sample size 7 shells. Shell Length 29.6–38.1 mm mean L/H = 1.4, mean L/B = 2.0

***Morphotype 4*** (Figure 8). Outline ovate–elongate. White or cream shells with black blotches over lunule and escutcheon. Sample size 75 shells. Shell Length 20.7–39.3 mm, mean L/H = 1.4 , mean L/B = 2.4 .

***Morphotype 5*** (Figure 9). Outline ovate-elongate. Cream to beige shells with prominent darker anastomosing radial zigzag streaks. Sample size 38 shells. Shell Length 12.4–43.2 mm, mean L/H = 1.4, mean L/B = 2.4.

####### Distribution.

The type locality is recorded as Tuticorin on the Coromandel coast (Chemnitz 1795). The species was subsequently recorded in various localities from Karachi to the Philippines (Ray 1948). Huber (2010) records this species from the east and west coasts of India, Andaman Sea, Indonesia, Philippines, South China, Singapore, Thailand, and Hainan. Records from the northern Arabian Sea may be *Marciacordata* (Forsskål in Niebuhr, 1775) and the most easterly confirmed records are from Gujarat. In India *Marciarecens* is recorded from the states of Goa, Gujarat, Karnataka, Kerala, Maharashtra, Odisha, and Tamil Nadu (Ramakrishna and Dey 2010, Subba Rao 2017) as a commonly available, commercially exploited edible clam (Alagarswami and Narasimham 1973, Durve 1975, Narasimham 1991). The presence of this species in estuaries was recorded by Ray (1948), Huber (2010), and Pati and Panigrahy (2013).

####### Remarks.

The species was originally described by Chemnitz (1795) and made available as *Venusrecens* by Holten (1802). *Venusmarmorata* Lamarck, 1818 is generally regarded as a synonym (Fischer-Piette and Metivier 1971; Huber 2010) despite the type locality given by Lamarck as ‘southern Europe’. Examination of the type material in the Geneva Museum of Natural History (MHNG-MOLL-504213) supports the synonymy.

The morphotypes differ primarily in colour pattern with some variation in relative timidity as shown by the L/B ratios that vary from 2.0 to 2.4. This ratio is distinctly different from the 1.6 for *M.opima*. The sampling regime was not precise enough to distinguish if there was any relationship between morphotype and distribution.

###### Genus Protapes

Taxon classificationAnimaliaVeneridaVeneridae

Dall, 1902

####### Type species.

*Venusgallus* Gmelin, 1791

####### Description.

Moderately large, outline triangular ovate to oval, often posteriorly truncated and pronounced anteriorly; lunule margin excavated. Hinge with three cardinal teeth in each valve. Ligament external, opisthodetic. Pallial sinus steeply ascending towards the umbonal cavity. External sculpture strong, of raised commarginal ridges. External patterns predominantly of interrupted zig-zag lines.

####### Remarks.

The genera *Protapes*, *Paphia*, and *Paratapes* all share the character of the ascending pallial sinus. *Paphia* and *Paratapes* differ in outline and sculpture in being elongate, distinctly longer than high, and having a smooth shell.

Three species of *Protapes* are recorded from Indian waters, *P.gallus* (Gmelin, 1791), *P.ziczac* (Linnaeus, 1758), and *P.monstrosus* (Römer, 1870), and all are well illustrated by Huber (2010). Only *P.gallus* and *P.ziczac* were collected in this study.

###### Protapes gallus

Taxon classificationAnimaliaVeneridaVeneridae

(Gmelin, 1791)

Figure 10

####### Original combination.

*Venusgallus* Gmelin, 1791

####### Synonyms.

(from MolluscaBase 2018a) *Venusmalabarica* Dillwyn, 1817; *Venusrhombifera* Bory de Saint-Vincent, 1827; *Tapeslentiginosa* Reeve, 1864.

####### Type locality.

As the name *Venusmalabarica* Chemnitz, 1782 is unavailable this species takes the name of *Venusgallus* Gmelin, 1791, both names referring to Chemnitz, 1782 tab. 31, figs 324–325. The type locality is given as the Malabar coast by Chemnitz (1782), which largely equates with the coast of modern Kerala.

####### Material examined.

Neendakara, Kollam, 5 live collected specimens + 10 empty articulated shells; Dharmadam, Kannur, 6 live collected specimens + 4 empty articulated shells.

####### Description.

Shell to 60 mm in length, solid, compressed, inequilateral, beaks slightly to the anterior. Outline trigonal-subovate, lunule margin impressed, anterior margin pronounced, posterior ventral margin weakly truncated, posterior dorsal margin sloping steeply. Lunule lanceolate, demarcated by shallow groove. Escutcheon long, narrow, weakly striated. Sculpture of evenly sized, rounded, closely spaced, commarginal ribs separated by narrow grooves. Pallial sinus wide, deep, ascending steeply toward umbonal cavity. External colouration light brown with narrow and light zigzag or chevron streaks with four distinct brown rays radiating from the beak to the ventral margin. Shell interior white with yellowish tinge on the umbonal cavity.

####### Distribution.

*Protapesgallus* has an Indo-West Pacific distribution extending from India to China (Huber 2010) but Chen et al. (2014) suggests that cryptic species may also be present.

####### Remarks.

This species was described as *Venusmalabarica* by Chemnitz (1782, figs. 324, 325) with the type locality of the Malabar coast in southwest India. Using Chemnitz’s (1782) figures, Gmelin (1791) erected the name *Venusgallus* and repeated the type locality of the Malabar coast. No type material could be located in Copenhagen or St Petersburg collections, consequently all nomenclature is based on the figures in Chemnitz. These figures are sufficient to place this taxon in the genus *Protapes* and this taxon was adopted as the type of the genus by Dall (1902). Although Chemnitz’s name is invalid for nomenclatural purposes, it was still being used in the late twentieth century by Fischer-Piette and Metivier (1971).

**Figure 10.**
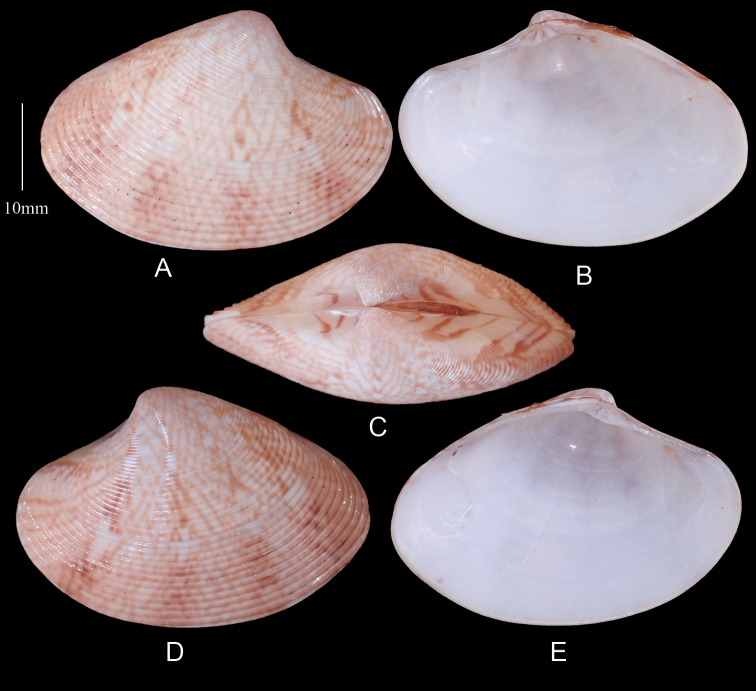
*Protapesgallus* Topotype **A** right valve dorsal **B** right valve ventral **C** dorsal margin **D** left valve dorsal **E** left valve ventral (DABFUK), Dharmadam, Kerala, South west coast of India.

###### Protapes ziczac

Taxon classificationAnimaliaVeneridaVeneridae

(Linnaeus, 1758)

Figure 11

####### Original combination.

*Venusziczac* Linnaeus, 1758

####### Synonyms.

(from MolluscaBase 2018d) *Venussinuosa* Lamarck, 1818; *Tapesinflata* Römer, 1870

####### Type locality.

Linnaeus (1758) gives the type locality as the Indian Ocean.

####### Material examined.

Neendakara, Kollam, 5 live collected specimens + 18 empty articulated shells; Dharmadam, Kannur, 3 live collected specimens.

####### Description.

Shells to 50 mm, solid, inflated, equivalve, inequilateral, beaks slightly in front of midline. Outline sub-ovate, lunule margin impressed, anterior margin pronounced, posterior ventral margin subtruncate, posterior area strongly sinuous. Lunule lanceolate, weakly ridged. Escutcheon narrow, smooth. Sculpture of raised concentric ridges separated by nearly equal-sized grooves. Pallial sinus narrow, apex rounded, ascending steeply towards umbonal cavity. Shell external colour tan with bright zigzag streaks and four brown rays radiating from umbo to ventral margin. Shell interior colour white with yellowish tinge in umbonal cavity.

####### Distribution.

The species has an Indian Ocean distribution with records from the Red Sea, Aden, East Africa, Somalia, Mozambique, Maputo, Inhambane, Nacala, Natal, Madagascar, Oman, and Persian Gulf (Huber 2010).

####### Remarks.

*Protapesziczac* (Linnaeus, 1758) has an inflated, heavy, and solid shell with the external sculpture the strongest of any species of *Protapes*.

The species was recorded as *Protapessinuosa* (Lamarck 1819) by Oliver and Glover (1996) from the Arabian Sea, but has subsequently been shown to be *Protapesziczac* (Huber, 2010). Huber (2010) gave *P.sinuosa* as a junior synonym and also synonymised *Tapesinflata* Römer, 1870 with *P.ziczac*, but Huber (2010) doubted the locality given as Sri Lanka. He regarded the Indo-Pacific shells identified as *P.sinuosa* as a new taxon, *P.swenneni* (Huber, 2010).

**Figure 11.**
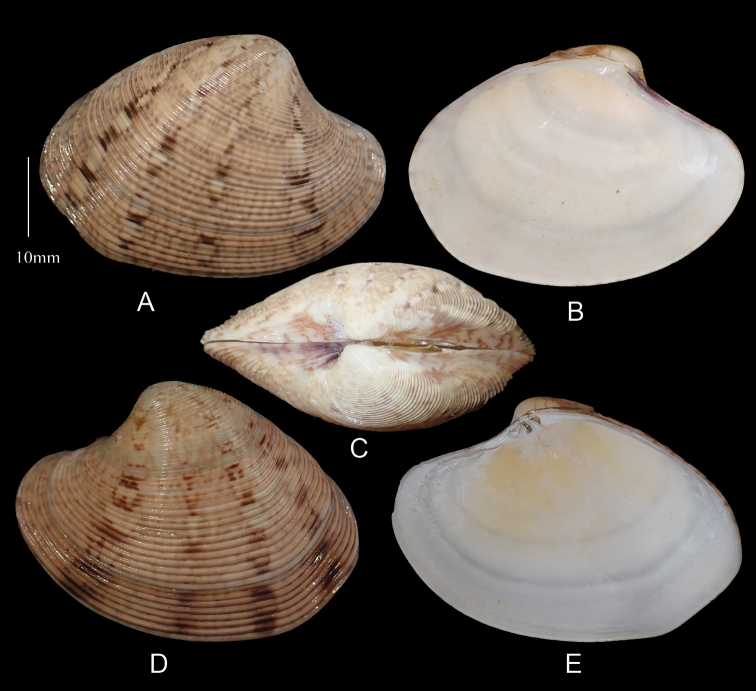
*Protapesziczac***A** right valve dorsal **B** right valve ventral **C** dorsal margin **D** left valve dorsal **E** left valve ventral (DABFUK), Kollam, Kerala, South west coast of India.

#### Discussion

It is evident from the results that the Ashtamudi Lake short-neck clam fishery is based primarily on *Marciarecens* and not *Paphiamalabarica* (= *Protapesgallus*). It is surprising that two such different shells should have become so confused. The literature citations for *Paphiamalabarica* in India are extensive (Ramakrishna and Dey 2010) but many of them are not accompanied by illustrations and therefore they do not allow identification. Where illustrations are presented as *Protapes* (Subba Rao et al. 1987: pl VII, fig. 12), Subba Rao et al. (1992: pl 26, fig. 1), and Ramakrishna and Dey (2010: pl XLIX, fig. 247), the generic identifications are correct. That, illustrated by Ramakrishna and Dey (2010: pl XLIX, fig. 247) seems to resemble *P.monstrosus* due to its more solid and inflated shell. Ramakrishna and Dey (2010: pl XLIX, fig. 247) and Subba Rao (2017: pl 77, fig. 352) have clearly illustrated the characteristic ascending pallial sinus of the genus *Protapes*. A similar historical background for *Marciarecens* also exists again exemplified by the references cited in Ramakrishna and Dey (2010). In this case no accompanying illustrations were present.

It would appear that a lack of illustrations in recent Indian literature have led to a misidentification and this has been carried into modern references referring to the Ashtamudi clam even where *Marcia* shells are illustrated: Appukuttan (1993: pl 1, fig. A, B, pl II), Joe (1993: 39, photo 2), CMFRI (2006: 78), Kripa et al. (2006: 9; fig. 1), Mohamed et al. (2013: 16, 20), Smita (2014: pl1.4 a, b, c, pl 1.5), Ampili (2014: 8, pl 1.1, fig. 1.1a; 161, pl, 7.1, fig. 7.2a), Ampili and Sreedhar (2015: 2, fig. 1), and CMFRI (2015, cover page). The taxonomic errors in identification in the initial publications were further exacerbated by subsequent publications, including those of the Central Marine Fisheries Research Institute in India. This has also resulted in the certification of fishery in the name of *Paphiamalabarica* by the Marine Stewardship Council in 2014. The images shown in the websites of Central Marine Fisheries Research Institute in India, World Wide Fund for Nature India and Marine Stewardship Council related to certification of Ashtamudi Lake clam fishery also present images of *Marcia* spp. as *Paphiamalabarica*.

No deleterious effects on the viability of the fishery have resulted from this error in identification but from a legislative context applying the incorrect name to the exploited species could undermine its certification and protection. On the basis of this study, the species involved in the Marine Stewardship Council certification would be better considered at the generic level of *Marcia* or at the species level for *Marciarecens*, the most dominant species in the Ashtamudi Lake clam fishery zone. We have limited this proposal to the Ashtamudi fishery as, at this time, we are unable to confirm the identity of clams from other fisheries, including those cited as exploiting *Marciaopima*. It is possible that the name “short-neck clam” is applied loosely to both *Marcia* species. Subba Rao gives the Tamil name “vazhukku matti” for *M.opima* but none for *M.recens*, perhaps indicating a lack of discrimination by fishermen. In this context it will be necessary to ascertain the relative abundance of the two *Marcia* species in any fishery and elucidate the ecology of these species especially their micro-habitat preferences.

Misidentification can undermine comparative biological studies. For example, Joy and Chakraborty (2017) describe anti-oxidant properties extracted from the Ashtamudi clams but wrongly identify them as *Paphiamalabarica (= Protapesgallus)*. Any subsequent attempts to repeat such research using true *Protapes* may give entirely different results.

*Marciarecens* has now been confirmed from its type locality in Tuticorin and is conspecific with the Ashtamudi Lake population in Kerala. Similar shells are known from further north at Mumbai but its northern limit is not known nor where, or if, it overlaps with *Marciacordata*, a widespread Arabian species. It would appear that *M.recens* extends throughout the Indo-Pacific although the molecular data from Chen et al. (2011) suggests that the systematics of *Marcia* may be more complex than currently estimated.

While this study has correctly identified the Ashtamudi clam, many unresolved issues surround this species and the genus as a whole. Molecular studies are necessary to resolve the population differences within *M.recens* and the relationship between it and *M.cordata* and *M.opima*. *Marciarecens* is in many ways similar to some species of *Tapes* and *Politapes* and here too a molecular resolution is needed. Morphologically other species of *Marcia* have been separated into *Hemitapes* (Huber 2010) and this too needs a molecular clarification.

## Supplementary Material

XML Treatment for Marcia

XML Treatment for Marcia opima

XML Treatment for Marcia recens

XML Treatment for Protapes

XML Treatment for Protapes gallus

XML Treatment for Protapes ziczac

## References

[B1] Achari GPK (1986) Investigations on ecophysiologlcal factors influencing developmental biology of clams. Central Marine Fisheries Research Institute, India (CMFRI) Annual Report 1985–1986, 61 pp.

[B2] Adams H Adams A (1857) The genera of Recent Mollusca; arranged according to their organization. London, van Voorst. [Published in parts: Vol. 3, pl. 113–128.]

[B3] Alagarswami K Narasimham KA (1973) Clam, cockle and oyster resources of the Indian coasts. Central Marine Fisheries Research Institute, India Special Publication, 648–658.

[B4] Ampili M (2014) Adaptability, Distribution status and phylogeny of selected venerid clams. PhD Thesis, Mahatma Gandhi University, Kerala.

[B5] Ampili M Sreedhar SK (2015) Morphotypes: Morphological plasticity in *Paphiamalabarica* (Chemnitz) (Mollusca: Bivalvia) of a deep estuary, Ashtamudi estuary. International Journal of Scientific and Research Publications 5(6): 1–4.

[B6] Appukuttan KK (1993) Studies on the ecobiology and fishery of *Paphiamalabarica* (Chemnitz) (Veneridae, Bivalvia) from Ashtamudi estuary, south west coast of India. PhD Thesis, University of Kerala, Kerala.

[B7] Appukuttan KK (2016) Ashtamudi clam fishery – 1^st^ MSG Certified fishery in India. In: Nandan NS, Oliver GP, Jayachandran RR, Asha CV (Eds) Training manual, 1^st^ International training workshop on taxonomy of bivalve molluscs, Directorate of Public Relations and Publications, Cochin University of Science and Technology, Cochin, 54–64.

[B8] Appukuttan KK Aravindan CM Yohanan TM Balasubramanian NK (1999) Population dynamics of an exploited stock of the clam *Paphiamalabarica* of Ashtamudi estuary (South India). In: Fourth Indian Fisheries Forum, 1996, School of Marine Sciences, Cochin University of Science and Technology, Cochin, 31–34.

[B9] Appukuttan KK Thomas KT Joseph M Nair TP (1985) Baby clam (*Katelysiaopima*) fishery in Ashtamudi backwaters. Journal of the Marine Biological Association of India 27(1, 2): 15–20.

[B10] Chemnitz JH (1782) Neues systematisches Conchylien-Cabinet. Sechster Band. Mit sechs und dreyßig nach der Natur gemalten und durch lebendige Farben erleuchteten Kupfertafeln. Nürnberg. Raspe, 375 pp.

[B11] Chemnitz JH (1795) Neuessystem atischen Conchylien Cabinet. vol. 11. Gabriel Nicolaus Raspe, Nürnberg, 310 pp 10.5962/bhl.title.120155

[B12] Chen J Li Q Kong L Yu H (2011) How DNA barcodes complement taxonomy and explore species diversity: the case study of a poorly understood marine fauna. PLoS ONE 6(6), e21326. 10.1371/journal.pone.0021326 PMC3116896 21698181

[B13] Chen J Li Q Zhang S-P Kong L-F Wang X-L (2014) Additional lines of evidence provide new insights into species diversity of the PaphiasubgenusProtapes (Mollusca, Bivalvia, Veneridae) in seas of south China. Marine Biodiversity 44(1): 55–61. 10.1007/s12526-013-0184-1

[B14] CMFRI (1988) Population studies on clam resources. Central Marine Fisheries Research Institute, India, Annual Report, 24 pp.

[B15] CMFRI (2006) CMFRI Annual Report 2005–2006. Central Marine Fisheries Research Institute, Cochin, India, 141 pp.

[B16] CMFRI (2011) CMFRI Annual Report 2010–2011. Central Marine Fisheries Research Institute, Cochin, India, 163 pp.

[B17] CMFRI (2015) CMFRI Annual Report 2014–15. Central Marine Fisheries Research Institute, Cochin, India, 353 pp.

[B18] CMFRI (2017) Annual Report 2016–17. Central Marine Fisheries Research Institute, Cochin, India, 292 pp.

[B19] Dall WH (1902) Synopsis of the family Veneridae and of the North American recent species. Proceedings of the United States National Museum 26: 335–412. 10.5479/si.00963801.26-1312.335

[B20] Dillwyn LW (1817) A descriptive catalogue of Recent shells, arranged according to the Linnean method, with particular attention to the synonymy. John and Arthur Arch, Cornhill, London, 580 pp [Vol. 1], 512 pp [Vol. 2].

[B21] Durve VS (1975) Commercial marine molluscs of India and the need for their survey. Records of Zoological Survey of India 68: 421–429.

[B22] Fischer-Piette E Metivier B (1971) Revision des Tapetinae (Mollusques bivalves) Memoirs du MNHN, Paris, ser. A zoologie 71: 1–106.

[B23] Forsskål P (1775) Descriptiones animalium avium, amphibiorum, piscium, insectorum, vermium quae in itinere orientali observavit Petrus Forskål, prof. Haun., post mortem auctoris edidit Carsten Niebuhr. Hauniae [Copengagen], Möller, 1–19 + i-xxxiv + 1–164, 1 map.

[B24] Gmelin JF (1791) Caroli a Linne, Systemae naturae ed. 13. Auctareformata VermesTestacea 1(6): 3021–3910.

[B25] Gray JE (1851) List of the specimens of British animals in the collection of the British Museum. Part 7, Mollusca Acephala and Brachiopoda. British Museum, London, 167 pp.

[B26] Holten HS (1802) Anmaerkininger till Beskrivelsen over Zeus guttatussamt Beskrivelser over tvendenye Arter Lernaer Skrivter af Naturhistorie-Selskabet 5(2): 129–137. [Tab. II [= 3]. Kiøbenhavn]

[B27] Huber M (2010) *Compendium of Bivalves*. Conch Books, Hackenheim, 901 pp.

[B28] Joe OW (1993) Distribution of trace metals in Ashtamudi Estuary, Kerala, with special reference to the Molluscs. PhD Thesis, University of Kerala, Kerala.

[B29] Joy M Chakraborty K (2017) An unprecedented antioxidative isopimarane norditerpenoid from bivalve clam, *Paphiamalabarica* with anti-cyclooxygenase and lipoxygenase potential. Pharmaceutical Biology 55: 819–824, 10.1080/13880209.2017.1280061 PMC6130755 28116944

[B30] Kripa V Sreejaya R Shiju AA Radhakrishnan P Swarnalatha P Anasu Koya A Mohamad KS Mutiah P (2006) Remote setting of the yellow clam *Paphiamalabarica* and the pearl oyster *Pinctadafucata* in India. Marine Fisheries Information Service, Central Marine Fisheries Research Institute, India 190: 8–13.

[B31] Lamarck JBM de (1818) Histoire naturelle des animaux sans vertèbres. Tome cinquième. Deterville/Verdière, Paris, 612 pp.

[B32] Linnaeus C (1758) Systema naturæ per regna tria naturæ, secundum classes, ordines, genera, species, cum characteribus, differentiis, synonymis, locis. Tomus I. Editio decima, reformata, Holmiæ. (Salvius) 1758(1–4): 1–824.

[B33] Mohamed KS Venkatesan V Kripa V Prema D Mathew Joseph Alloycious PS Jenny Sharma Valsala KK Saji Kumar KK Ragesh N John Bose Anjana Mohan (2013) Fishery Management Plan for Ashtamudi Lake Clam Resources. Central Marine Fisheries Research Institute, India, Special Publication No 114, 48 pp.

[B34] MolluscaBase (2018a) *Protapesgallus* (Gmelin, 1791) Accessed through: World Register of Marine Species. http://www.marinespecies.org/aphia.php?p=taxdetails&id=507880 [2018-08-15]

[B35] MolluscaBase (2018b) *Marciaopima* (Gmelin, 1791). Accessed through: World Register of Marine Species. http://marinespecies.org/aphia.php?p=taxdetails&id=507769 [2018–08–15]

[B36] MolluscaBase (2018c) *Marciarecens* (Holten, 1802). Accessed through: World Register of Marine Species. http://marinespecies.org/aphia.php?p=taxdetails&id=507770 [2018–08–15]

[B37] MolluscaBase (2018d) *Protapesziczac* (Linnaeus, 1758). Accessed through: World Register of Marine Species. http://marinespecies.org/aphia.php?p=taxdetails&id=507881 [2018–08–15]

[B38] Nandan SB Oliver PG Jayachandran RR Asha CV (Eds) (2016) Training manual, 1^st^ International training workshop on taxonomy of bivalve molluscs, Directorate of Public Relations and Publications, Cochin University of Science and Technology, Cochin, 349 pp.

[B39] Narasimham KA (1991) Present status of clam fisheries of India. Journal of Marine Biological Association of India 30: 76–88.

[B40] Oliver PG (1992) Bivalved Seashells of the Red Sea. National Museum of Wales, Cardiff, 330 pp.

[B41] Oliver PG Glover E (1996) Paphia (Protapes) (BivalviaVeneroidea) in the Arabian Sea with the description of a new species. Journal of Conchology 35: 389–405.

[B42] Pati P Panigrahy R (2013) On some mollusca collections from different beaches of south Odisha coast of India. Records of Zoological Survey of India 113: 229–254.

[B43] Rafinesque CS (1815) Analyse de la Nature ou Tableau de l’Univers et des Corps organises. Palerme, 1–224.

[B44] Ramakrishna Dey A (2010) Annotated Checklist of Indian Marine Molluscs (Cephalopoda, Bivalvia and Scaphopoda): Part-1. Records of Zoological Survey of India (Occasional) Paper No. 320: 1–357.

[B45] Ray HC (1948) On a collection of Mollusca from the Coromandel coast of India. Records of the Indian Museum 47: 87–122.

[B46] Römer E (1864) Beschreibung neuer Arten von *Venus*. Malakozoologische Blätter 11: 119–123.

[B47] Römer E (1870–1872) Monographie der Molluskengattung *Venus* Linné. – Novitates Conchologicae Supplement 3: 1–128. [Taf. I-XL [= 1–40]. Cassel]

[B48] Subba Rao NV (2017) Indian Seashells, Part B Bivalvia. Zoological Survey of India, Kolkata, 676 pp.

[B49] Subba Rao NV Dey A Barua S (1992) Estuarine and Marine Mollluscs. Fauna of West Bengal, State Fauna Series, Zoological Survey of India 3: 129–268.

[B50] Subba Rao NV Surya Rao KV Mitra SC (1987) Malacological notes on Sagar island. Bulletin of Zoological Survey of India 8(1–3): 149–158.

[B51] Smita SN (2014) Studies on ecobiology of *Paphiamalabarica* (Chemnitz) from estuarine habitats of Goa. PhD Thesis, CSIR-National Institute of Oceanography and Goa University, Goa.

[B52] Wakamatsu M Wakamatsu H (2017) The certification of small-scale fisheries. Marine Policy 77: 97–103. 10.1016/j.marpol.2016.12.016

